# Comparison of craniotomy and decompressive craniectomy for acute subdural hematoma: a meta-analysis of comparative study

**DOI:** 10.1097/JS9.0000000000001590

**Published:** 2024-05-13

**Authors:** Hua Li, Yuqiang Yao, Yuwen Jiang, Yibing Su, Hanbin Wang, Can Zhu, Wenyi Gan

**Affiliations:** a Department of Orthopedics, Beijing Jishuitan Hospital; b Department of Neurosurgery, Beijing Jishuitan Hospital, Beijing; c Department of Traumatology, Zhuhai People’s Hospital, Zhuhai, Guangdong; d Second Department of Clinical Medicine, Anhui Medical University, Hefei, Anhui, People’s Republic of China

**Keywords:** acute subdural hematoma, comparative studies, craniotomy, decompressive craniectomy, meta-analysis, outcomes

## Abstract

**Background:**

Acute subdural hematoma (ASDH) necessitates urgent surgical intervention. Craniotomy (CO) and decompressive craniectomy (DC) are the two main surgical procedures for ASDH evacuation. This meta-analysis is to compare the clinical outcomes between the CO and DC procedures.

**Materials and methods:**

The authors performed a meta-analysis according to Preferred Reporting Items for Systematic reviews and Meta-Analyses (PRISMA, Supplemental Digital Content 1, http://links.lww.com/JS9/C513, Supplemental Digital Content 2, http://links.lww.com/JS9/C514) Statement protocol and assessing the methodological quality of systematic reviews (AMSTAR) (Supplemental Digital Content 3, http://links.lww.com/JS9/C515) guideline. The PubMed, Embase, Web of Science, and Cochrane Library databases were systematically searched. Comparative studies reporting the outcomes of the CO and DC procedures in patients with ASDH were included.

**Results:**

A total of 15 articles with 4853 patients [2531 (52.2%) receiving CO and 2322 (47.8%) receiving DC] were included in this meta-analysis. DC was associated with higher mortality [31.5 vs. 40.6%, odds ratio (OR)=0.58, 95% CI: 0.43–0.77] and rate of patients with poorer neurological outcomes (54.3 vs. 72.7%; OR=0.43, 95% CI: 0.28–0.67) compared to CO. The meta-regression model identified the comparability of preoperative severity as the only potential source of heterogeneity. When the preoperative severity was comparable between the two procedures, the mortality (CO 35.5 vs. DC 38.1%, OR=0.80, 95% CI: 0.62–1.02) and the proportion of patients with poorer neurological outcomes (CO 64.8 vs. DC 66.0%; OR=0.82, 95% CI: 0.57–1.16) were both similar. Reoperation rates were similar between the two procedures (CO 16.1 vs. DC 16.0%; OR=0.95, 95% CI: 0.61–1.48).

**Conclusion:**

Our meta-analysis reveals that DC is associated with higher mortality and poorer neurological outcomes in ASDH compared to CO. Notably, this difference in outcomes might be driven by baseline patient severity, as the significance of surgical choice diminishes after adjusting for this factor. Our findings challenge previous opinions regarding the superiority of CO over DC and underscore the importance of considering patient-specific characteristics when making surgical decisions. This insight offers guidance for surgeons in making decisions tailored to the specific conditions of their patients.

## Introduction

Highlights

- Overall, decompressive craniectomy seemed to be associated with higher mortality and poorer neurological outcomes in evacuating acute subdural hematoma as compared with craniotomy based on published studies.
- When baseline severity was adjusted to be comparable, the choice between craniotomy and decompressive craniectomy did not significantly impact patient outcomes.

Acute subdural hematoma (ASDH) represents a critical neurosurgical emergency usually resulting from traumatic head injuries, with reported mortality ranging from 40 to 60%^1–3^. Surgery is often mandatory to evacuate the hematoma and relieve elevated intracranial pressure (ICP).

Craniotomy (CO) and decompressive craniectomy (DC) serve as the two main surgical procedures recommended by guidelines for ASDH evacuation. CO entails a removal of a part of the skull, clearance of the subdural hematoma, and subsequent replacement of the skull flap^4^. DC, a more invasive option, intentionally leaves the skull flap open to prevent future ICP increase. Primary DC is usually performed in cases of severe brain swelling or as a preventive measure^5^. Theoretically, DC provides superior ICP control but its long-term benefits, especially in preventive contexts, are still controversial. In addition, DC is often related to a higher incidence of complications, such as postoperative infections^6^. The Brain Trauma Foundation guideline for surgical treatment of ASDH does not offer the optimal indication between the two approaches^7^.

Several studies have investigated the comparative efficacy and safety of the two approaches in patients with ASDH. Their findings remain inconclusive. Woertgen *et al*.^8^ reviewed the data of 180 patients with ASDH undergoing DC or CO and found higher mortality in the DC group compared to the CO group (53 vs. 32.3%). The authors attributed this result to a higher proportion of patients experiencing herniation within the DC group. In a national cohort analysis by Rush *et al*.^9^, the DC group continued to exhibit higher mortality than the CO group (32 vs. 22%), even after adjustment of preoperative baseline characteristics using the propensity score matching. Nevertheless, a recent randomized controlled trial (RCT) involving 450 patients reported comparable mortality between the two procedures, with a noteworthy increase in reoperation rate observed in the CO group^10^.

The evaluation of CO and DC regarding their comparative efficacy and safety are essential considerations for surgeons. Given the uncertain evidence, investigating the benefits, and risks of CO and DC is still a subject of interest. Thus, we perform this meta-analysis to compare the outcomes of these two interventions in the context of ASDH.

## Materials and methods

This meta-analysis was conducted following the Preferred Reporting Items for Systematic reviews and Meta-Analyses (PRISMA) Statement protocol and AMSTAR (Assessing the methodological quality of systematic reviews) guideline^11,12^.

### Search strategy

We searched the PubMed, Embase, Web of Science, and Cochrane Library databases up to 1st October 2023. The search keywords were ‘decompressive craniectomy’ AND craniotomy AND (‘subdural hematoma’ OR ‘subdural hemorrhage’). MeSH Terms for decompressive craniectomy, craniotomy, and subdural hematoma were used. We developed specific search strategies for each database, and the references of the identified studies were checked for potential eligibility (SDC, Table 1 in the Supplement, Supplemental Digital Content 4, http://links.lww.com/JS9/C516).

### Eligibility criteria

We applied the following inclusion criteria to select eligible studies: (1) reporting outcomes of both CO and DC in patients with ASDH, and (2) employing a comparative study design (cohort studies and RCT). We excluded non-English language reports, *in vitro* studies, case reports, brief reports, conference abstracts/posters, and reviews. After the removal of duplicates, two authors reviewed the titles and abstracts to identify potentially eligible studies independently. Full texts were then assessed by the same two authors to identify the final list of papers suitable for inclusion in the current study. In cases of disagreement, a third senior doctor was consulted for a final consensus.

### Data extraction

We extracted the data on publication details, patient characteristics, operations, perioperative information, and study design. Recognizing the potential influence of baseline severity on outcomes, we addressed this by assessing baseline comparability based on the arriving Glasgow Coma Scale (GCS), which was set as an indicator of preoperative severity. A GCS below nine is generally classified as a severe coma. Studies with comparable arriving GCS between CO and DC groups were deemed to have comparable preoperative severity. The primary outcome of interest was the postoperative mortality. Neurological functional outcomes and reoperation were extracted as secondary outcomes. The poor functional prognosis was determined according to the following criteria: (1) Glasgow Outcome Scale (GOS) ≤3^13^, (2) Extended GOS ≤4^14^, or (3) modified Rankin Scale (mRS) ≥4^15^. The appendices of included papers were all reviewed for the potential data. If the necessary information could not be obtained from the original paper, we contacted the corresponding author to request additional information.

### Assessment of quality and bias

The quality of the included studies was assessed by two reviewers independently. In this regard, the modified Jadad Scale was used for RCT^16^, and the Newcastle–Ottawa Score (NOS) was used for cohort studies^17^. Publication bias was estimated using the funnel plot and Peters’ test^18^.

### Statistical analysis

We performed the statistical analysis using R software (version 4.1.3), with a significance threshold of *P*=0.05. For dichotomous variables like mortality, the odds ratios (ORs) with the 95% CIs were calculated using the Mantel–Haenszel (M–H) method. We used the *I* ^2^ statistic and *Q* test to assess the heterogeneity. If *I* ^2^ was less than 50% and the *P*-value for the *Q* test was greater than 0.05, the studies were considered to exhibit minimal heterogeneity. Generally, a random-effects model is employed to analyze the pooled results if *I* ^2^ exceeds 50% or the *P*-value for the *Q* test is below 0.05. Conversely, a fixed-effects model is employed if *I* ^2^ is less than 50% and the *P*-value for the Q test exceeds 0.05. In this meta-analysis, we preferred the random-effects model over the fixed-effects model considering the diverse study designs and potential variations of operative technique across included studies. Thus, a random-effects model was predetermined^19^. The sensitivity analysis was conducted using the leave-one-out analysis. We used the meta-regression method to explore the potential sources of heterogeneity based on the predetermined factors, including the year of publication, the mean age of patients, sample size, percentage of male participants, and comparability of preoperative baseline severity. Factors with a *P*-value of less than 0.1 in the regression model were extracted for subsequent subgroup analyses, and the heterogeneity of inter-subgroups and intra-subgroups was then evaluated.

## Results

### Overview of searching procedure

**Figure 1:**
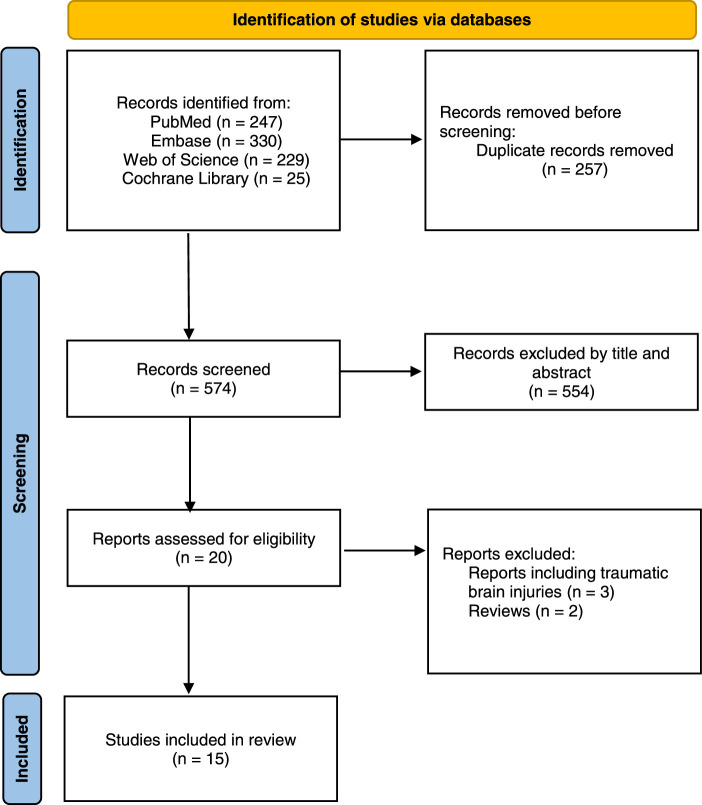
Preferred Reporting Items for Systematic Reviews and Meta-Analyses (PRISMA) flowchart of included studies.

The initial search yielded 831 studies. After removing duplicates, we scanned the titles and abstracts of 574 papers. Twenty papers were then assessed by full-text reading. Finally,15 papers were included in the final analysis (Fig. 1)^8–10,20–31^. Of the included studies, 12 were retrospective cohort studies^8,9,20–29,32,33^, two were prospective cohort studies^30,31^, and only one was an RCT^10^. Half of the papers were published within the last 5 years (Table 1).

**Table 1:** Features of included studies.

Authors	Year	Journal	Study design	Reported outcomes
Woertgen *et al*.^8^	2006	J Clin Neurosci	RC	Mortality, function, reoperation
Chen *et al*.^20^	2011	J Trauma	RC	Mortality, function, reoperation
Li *et al*.^21^	2012	Acta Neurochir (Wien)	RC	Mortality, function
Tsermoulas *et al*.^23^	2016	World Neurosurg	RC	Mortality, function, reoperation
Kwon *et al*.^22^	2016	Korean J Neurotrauma	RC	Mortality, reoperation
Rush *et al*.^9^	2016	World Neurosurg	RC	Mortality
Vilcinis *et al*.^23^	2017	World Neurosurg	RC	Mortality, function
Shibahashi *et al*.^24^	2019	J Neurosurg	RC	Mortality
Altaf *et al*.^25^	2020	Pak J Med Sci	RC	Mortality, function
Castaño-Leon *et al*.^29^	2020	J Neurosurg Sci	PC	Mortality, function, reoperation
Ahemed *et al*.^26^	2021	Am Surg	RC	Mortality
Anis *et al*.^27^	2022	Asian J Neurosurg	RC	Mortality, function, reoperation
Ruggeri *et al*.^28^	2022	J Neurosurg Sci	RC	Mortality
Hutchinson *et al*.^10^	2023	N Engl J Med	RCT	Mortality, function, reoperation
van Essen *et al*.^30^	2023	EClinicalMedicine	PC	Mortality, function, reoperation

PC, prospective cohort; RC, retrospective cohort; RCT, randomized controlled trial.

### Clinical characteristics

Overall, a total of 4853 patients were identified from the included studies, with 2531 (52.2%) receiving CO and 2322 (47.8%) receiving DC. The overall cohort displayed a male predominance (71.5%), and the average age ranged from 36.8 to 67 years. All but one study offered the data regarding the arriving GCS. Six studies showed comparable GCS between the groups, while the remaining eight studies reported a significantly higher GCS in the CO group. In sum, 70.4% of patients in the CO group had an arriving GCS below nine compared to 80.9% in the DC group. The only study without arriving GCS data employed propensity score matching to ensure a comparable preoperative baseline between the CO and DC groups. Nine studies provided the incidences of patients with signs of preoperative herniation (collapse of ventricles or cisterns, herniation, and/or severe midline shift), revealing higher prevalence in the DC group (62.8%) compared to CO (41.0%) (Table 2).

**Table 2:** The detailed information of clinical characteristics.

Authors	Sample size (CO/DC) (*n*)	Mean age (y)	Gender ratio (Male)	Mean PreGCS (CO/DC)	Ratio of PreGCS≤8 (CO/DC)	Ratio of herniation sign (CO/DC)	Comparability of severity	Death (CO/DC) (*n*)	Poor function (CO/DC) (*n*)	ReOP (CO/DC) (*n*)
Woertgen *et al*.^8^	111/69	55.2	63%	NA	60.4%/69.6%	31.5%/49.3%	DC severe	32/31	40/18	33/19
Chen *et al*.^20^	42/60	39.8	61%	6.3/5.9	100%/100%	42.9%/46.7%	Equal	3/14	26/33	6/11
Li *et al*.^21^	36/49	50.9	60%	9.5/5	38.9%/77.6%	19.4%/57.1%	DC severe	12/19	17/21	NA
Tsermoulas *et al*.^23^	30/67	45.2	81%	NA	46.7%/76.1%	NA	DC severe	6/26	19/28	10/19
Kwon *et al*.^22^	20/26	64.6	61%	NA	35.0%/61.5%	25.0%/50.0%	DC severe	1/13	12/6	4/3
Rush *et al*.^9^	151/151	52.7	71%	NA	NA	NA	Equal^a^	33/48	NA	NA
Vilcinis *et al*.^23^	394/249	58.1	63%	9.3/5.3	43.1%/84.3%	10.2%/33.3%	DC severe	79/134	218/37	NA
Shibahashi *et al*.^24^	514/514	63.5	69%	7/7	69.3%/66.5%	NA	Equal	214/201	NA	NA
Altaf *et al*.^25^	40/18	44.4	76%	5.7/5	100%/100%	NA	Equal	34/18	4/0	NA
Castaño-Leon *et al*.^29^	65/166	43.1	80%	NA	67.7%/88.0%	70.8%/91.6%	DC severe	22/66	31/48	7/31
Ahemed *et al*.^26^	518/518	41.8	88%	8.9/7.9	NA	56.3%/69.2%	Equal	18/24	NA	2/4
Anis *et al*.^27^	87/78	67	65%	9.6/7.9	44.0%/61.4%	NA	DC severe	26/24	NA	NA
Ruggeri *et al*.^28^	50/44	36.8	75%	3/3	100%/100%	NA	Equal	194/221	NA	NA
Hutchinson *et al*.^10^	228/222	48.5	79%	8/7.5	64.9%/65.8%	86.4%/86.5%	Equal	65/68	79/69	28/13
van Essen *et al*.^30^	245/91	56.3	72%	7/4	NA	43.3%/54.9%	DC severe	58/35	108/18	43/25

CO, craniotomy; DC, decompressive craniectomy; n, number; NA, not available; PreGCS, preoperative Glasgow Coma Scale; ReOP, reoperation; y, year.

a Propensity score matching was employed in this study to ensure comparable preoperative baseline between the CO and DC groups and thus, the comparability of severity was deemed ‘Equal’.

### Assessment of quality and bias

**Figure 2:**
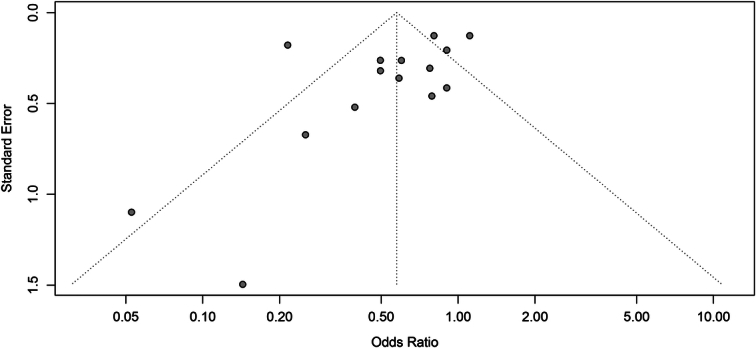
Funnel plot of the primary outcome (mortality).

The RCT had a modified Jadad score of seven, which indicated high-quality. The NOS for the cohort studies represented high-quality (Table 3). The funnel plot did not raise concerns of possible publication bias (Fig. 2), which was confirmed by the formal test (Peters’ test, *P*=0.140).

**Table 3:** Quality of included studies.

Research									
	Non-RCT / Newcastle–Ottawa Scale		Selection			Outcome			
	Representativeness of the exposed cohort	Selection of the nonexposed cohort	Ascertainment of exposure	Absence of the outcome of interest at the start	Comparability	Assessment of outcome	Adequate length of follow-ups	Accuracy of follow-up cohorts	Total
Woertgen *et al*.^8^	+	+	+	+	+	+	+	+	8
Chen *et al*.^20^	+	+	+	+	++	+	+	+	9
Li *et al*.^21^	+	+	+	+	+	+	+	-	7
Tsermoulas *et al*.^23^	+	+	+	+	+	+	+	+	8
Kwon *et al*.^22^	+	+	+	+	++	+	+	-	8
Rush *et al*.^9^	+	+	+	+	++	+	+	+	9
Vilcinis *et al*.^23^	+	+	+	+	+	+	+	+	8
Shibahashi *et al*.^24^	+	+	+	+	++	+	+	+	9
Altaf *et al*.^25^	+	+	+	+	+	+	+	+	8
Castaño-Leon *et al*.^29^	+	+	+	+	+	+	+	-	7
Ahemed *et al*.^26^	+	+	+	+	++	+	+	+	9
Anis *et al*.^27^	+	+	+	+	+	+	+	-	7
Ruggeri *et al*.^28^	+	+	+	+	++	+	+	+	9
van Essen *et al*.^30^	+	+	+	+	++	+	+	+	9
RCT / modified Jadad Score	Randomization		Concealment		Blinded		Withdraw or drop-out		Total
Hutchinson *et al*.^10^	2		2		2		1		7

### Primary outcome

**Figure 3:**
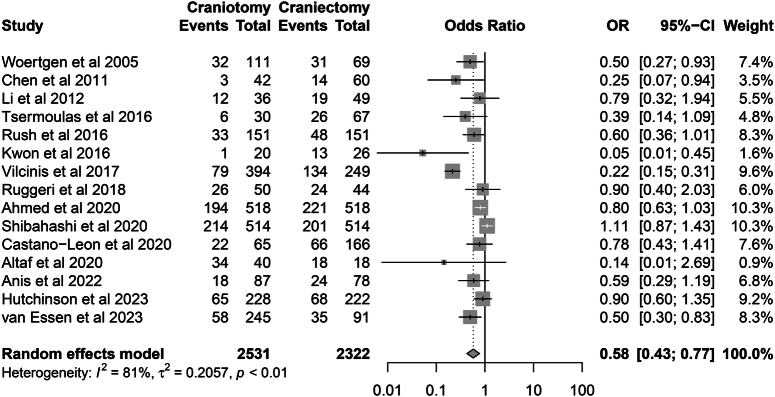
Forest plot of primary outcome (mortality).

**Figure 4:**
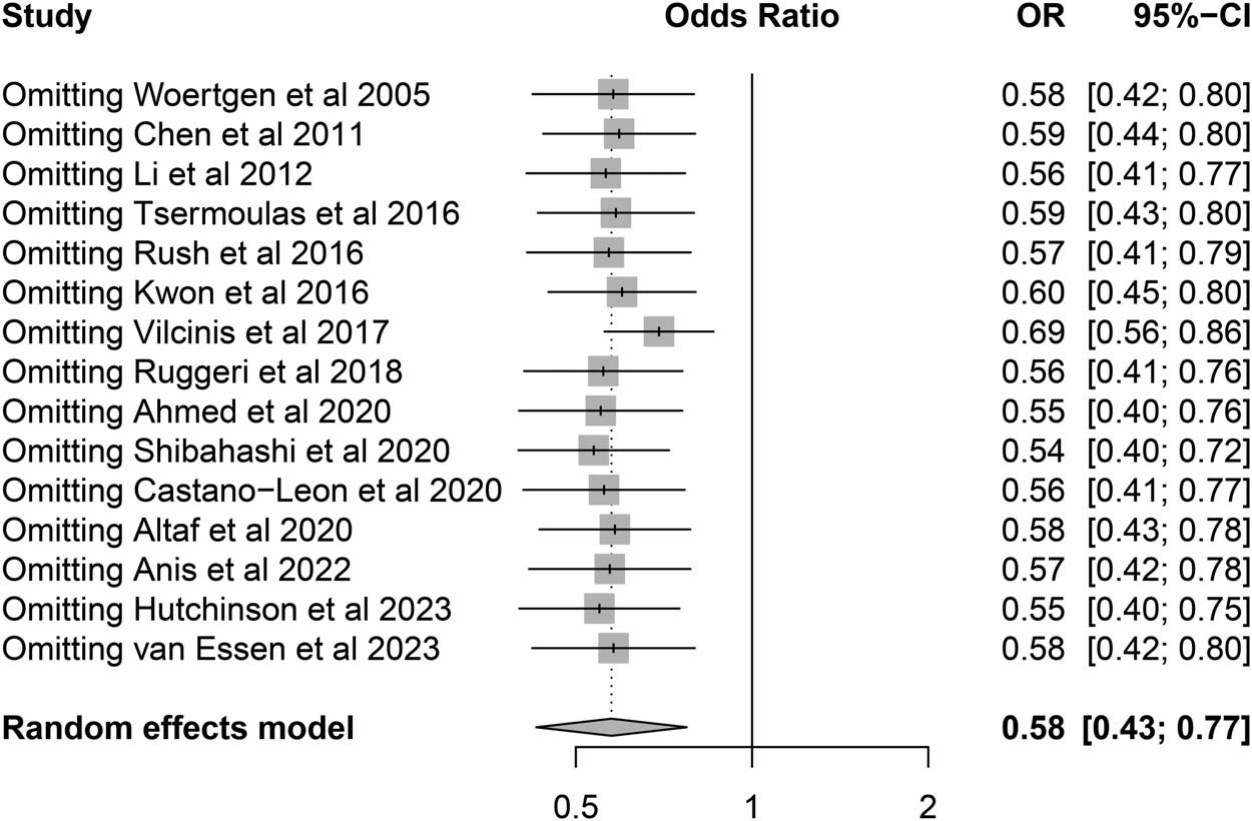
Sensitivity analysis of primary outcome (mortality) using the leave-one-out analysis.

All 15 studies reported the outcome of mortality. In total, there were 1739 deaths encountered in 4853 patients (35.8%). The pooled results showed that the DC group was associated with a higher risk of postoperative death than the CO group (Mortality: 31.5 vs. 40.6%; OR=0.58, 95% CI: 0.43–0.77, *P*=0.001), with considerable heterogeneity (*I* ^2^=81%, *P*=0.001) (Fig. 3). The pooled results were robust in the sensitivity analysis (Fig. 4).

**Figure 5:**
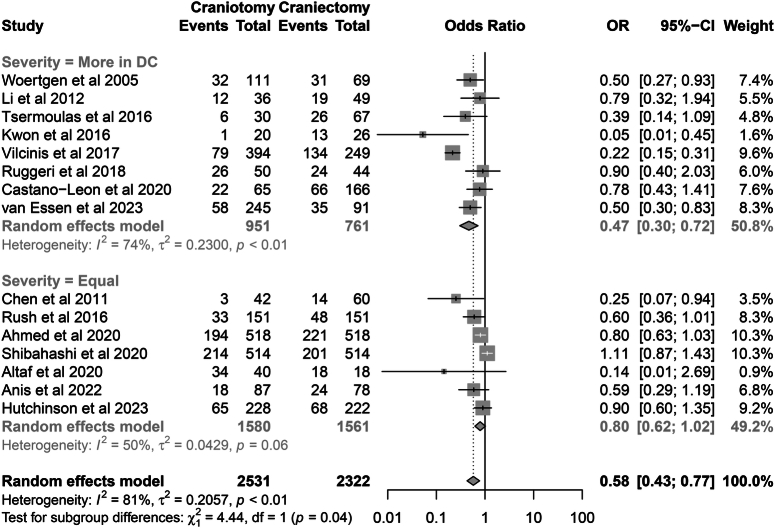
Subgroup analysis of mortality based on the comparability of preoperative severity.

The meta-regression model was performed to explore the source of heterogeneity. The comparability of preoperative baseline severity was identified to be a potential source of heterogeneity (Table 4) (SDC, Fig. 1 in the Supplement, Supplemental Digital Content 5, http://links.lww.com/JS9/C517). We further divided studies into two subgroups based on this factor. In the subgroup where the preoperative severity was similar, the mortality was 35.5% (561/1580) and 38.1% (594/1561) in patients undergoing CO and DC, respectively, which did not demonstrate statistical significance (OR=0.80, 95% CI: 0.62–1.02, *P*=0.070; Heterogeneity, *I* ^2^=50%, *P*=0.06). However, in the subgroup where the included studies had a lower arriving GCS in patients receiving DC, this procedure was associated with higher mortality compared to CO (24.8 vs. 45.7%; OR=0.47, 95% CI: 0.30–0.72, *P*=0.001; Heterogeneity, *I* ^2^=74%, *P*=0.01). Significant intra-subgroup heterogeneity was observed (*P*=0.035) (Fig. 5).

**Table 4:** Results of meta-regression model.

Factor	Coefficient (95% CIs)	*I* ^2^ in the model	*P*
Publication year	0.035 (−0.026–0.095)	75.3%	0.259
Mean age	−0.004 (−0.038–0.031)	74.0%	0.854
Percentage of male participants	2.3198 (−1.261–5.901)	73.5%	0.204
Sample size	0.001 (−0.001–0.001)	74.4%	0.248
Comparability of preoperative severity	0.4609 (−0.062–0.984)	67.2%	0.084

### Secondary outcomes

**Figure 6:**
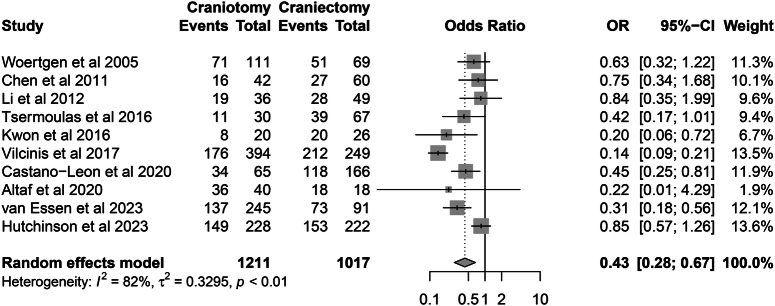
Forest plot of patients with poor prognosis.

Eleven studies reported data on neurological functional outcomes^8,10,20–26,28,30,31^, and the data of ten studies could be further combined. The pooled analysis showed that the proportion of patients with a poor functional prognosis was higher in the DC group (54.3 vs. 72.7%; OR=0.43, 95% CI: 0.28–0.67, *P*=0.001; Heterogeneity, *I* ^2^=82%, *P*=0.001) (Fig. 6). The study by Anis *et al*.^28^ reported that the mean value of postoperative GOS was 4.3 in both groups, with no statistical difference. The other four studies did not report the data regarding functional outcomes.

**Figure 7:**
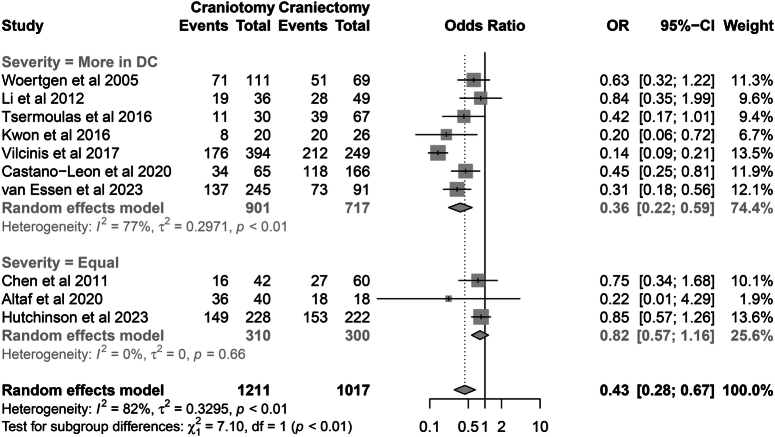
Subgroup analysis of neurological functional prognosis based on the comparability of preoperative severity.

The subgroup analysis showed that in studies where the preoperative severity was comparable between the two procedures, the incidence of patients with a poor prognosis was similar (64.8 vs. 66.0%; OR=0.82, 95% CI: 0.57–1.16, *P*=0.254; Heterogeneity, *I* ^2^=0, *P*=0.66). Conversely, in studies where the preoperative GCS was lower in the DC group, the aforementioned incidence was higher in the DC group (50.6 vs. 75.5%; OR=0.36, 95% CI: 0.22–0.59, *P*=0.001; Heterogeneity, *I* ^2^=77%, *P*=0.01) (Fig. 7).

**Figure 8:**
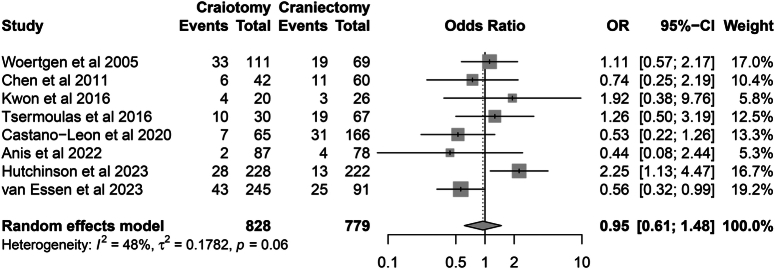
Forest plot of reoperations.

Eight studies reported data on reoperation rates^8,10,20,22,23,27,30,31^. The pooled results revealed no difference in reoperation rates between the CO and DC groups (16.1 vs. 16.0%; OR=0.95, 95% CI: 0.61–1.48, *P*=0.823; Heterogeneity, *I* ^2^=48%, *P*=0.06) (Fig. 8).

## Discussion

ASDH presents a significant neurological challenge, frequently arising from traumatic brain injuries with mortality ranging from 40 to 60%^34^. Surgical intervention is usually imperative to evacuate hematoma and alleviate elevated ICP. The primary techniques for ASDH evacuation, CO and DC, have been subjects of prolonged debate regarding their optimal indications. In the United States, CO was used 10 times more frequently than DC for ASDH^9^, while nearly half of European neurosurgeons preferentially use DC in over half of their patients^35^. The substantial variability in clinical practices and the absence of well-established evidence underscore the clinical relevance of our current meta-analysis.

Our meta-analysis compared the efficacy and safety of CO and DC in ASDH patients. We found that DC was associated with higher mortality and poorer neurological outcomes compared to CO. The reoperation rate did not differ significantly between the procedures. Notably, the subgroup analysis indicated that when the preoperative severity was comparable, the outcomes tended to be similar, suggesting that patient selection and baseline severity may partially explain the observed differences.

By pooling data from 15 studies, our meta-analysis illustrated that CO was associated with a nearly 10% absolute risk reduction in mortality and a superior functional prognosis. Some researchers have attributed higher mortality observed with DC to factors such as axonal stretch and delayed intracranial hemorrhage owing to a persistent bony defect^27,36,37^. Two previous meta-analyses investigated the comparative effectiveness between CO and DC for ASDH. The meta-analysis by Phan *et al*., which incorporated the data from five studies, reported lower mortality in the CO procedure (OR=0.41). The proportion of patients with poor functional outcomes was also lower in the CO group^32^. Mahadewa *et al*.^33^ analyzed six studies, and their results also showed a 41% increased risk of death with DC. Our findings were partially consistent with their results. Compared to previous analyses, our research encompassed a relatively large number of 15 comparative studies, which allowed for a more comprehensive assessment. In addition, both aforementioned reviews acknowledged the potential bias from retrospective study designs and unadjusted baseline differences between patient groups. Indeed, we noted that most of the included studies were of a retrospective nature. There is a possibility of selection bias, as surgeons may preferentially choose DC for more severe cases, who are inherently predisposed to poorer prognoses. Thus, a stratified analysis was warranted, as the simplistic interpretation of the comparison of pooled mortality might not be appropriate.

In contrast to previous meta-analyses that failed to account for baseline severity, our study addressed this potential bias using the meta-regression model and subgroup analysis to evaluate the impact of surgical choice on outcomes across different patient subgroups. Our results revealed that the differences in preoperative severity between CO and DC correlated positively with the differences in postoperative mortality between the two procedures. This trend extended to functional prognosis as well. All of these suggested that when the baseline severity was equivalent, the influence of surgical choices on outcomes might be minimal. The benefits of two surgeries may be limited to the comprehensive evacuation of hemorrhage, and the control of brain swelling by removing the bone-flap may not impact the outcomes significantly. Our inference challenged the conclusions of some previous studies and reviews, suggesting that DC might not significantly increase mortality compared to CO for ASDH. The observed mortality advantage of CO in these prior studies could be attributed to selection bias, as patients in the CO group might had less severe presentations. This inference also aligned with the findings of the only RCT^10^. The mechanisms and explanations for our findings were not clearly understood, but they might be related to the biological and physiological effects of CO and DC on the brain. CO could preserve the integrity of the skull but might limit the expansion of the brain. DC could provide more effective decompression but potentially disrupt cerebral autoregulation and haemodynamics^38^, which could exacerbate brain edema and lead to the herniation of cranial parenchyma via the bone window. Nguyen *et al*. measured perioperative changes in brain volume in 38 patients with ASDH. They observed that in 15 cases (39.5%), the postoperative cerebral volume was decreased^39^. Given the reported association between lower arriving GCS and postoperative brain swelling after hematoma evacuation^39^, the authors recommended that severely injured patients could derive greater benefits from DC, while those with mild or moderate injuries might be more suitable candidates for CO. However, our subgroup analysis may offer new insights for clinical practice, suggesting that DC remains a viable option even for patients with mild swelling. This approach is particularly applicable in emergency situations, where assessment time is limited and surgeons often struggle to conduct a comprehensive multisystem evaluation quickly. In such cases, choosing DC can reduce the likelihood of human errors and severe consequences due to incomplete surgical assessment. Furthermore, selecting DC as an initial surgical intervention provides doctors with greater flexibility in managing ASDH, especially in situations where the patient’s condition is dynamically changing.

Our research indicated no difference in the reoperation rates between the two groups. This finding should be interpreted cautiously due to limited data availability in approximately half of the included studies. Similar results were reported by Phan *et al*.^32^ in their meta-analysis based on the data of four studies. Generally, the nature of reoperations differs between the two procedures. Reoperations in the CO group were often the secondary DCs performed to address progressive cerebral edema, while those in the DC group were more commonly linked to surgery-related complications, such as incision infections, subgaleal hemorrhage, hydrocephalus, and herniation through the cranial defect. Gooch *et al*.^40^ reported a complication rate of up to 34% after DC. Besides, DC inherently necessitated a secondary surgical procedure, ‘cranioplasty’, which could introduce further potential complications, such as bone-flap reabsorption. A deeper understanding of the specific complications associated with each procedure is crucial for implementing targeted perioperative management strategies. Future research should focus on collecting more detailed information. By employing such a personalized and data-driven approach, we can optimize patient outcomes regardless of the chosen surgical approach.

We noted several limitations in our meta-analysis. First, the methodology of the meta-analysis was associated with the possibility of unavoidable missing relevant studies. The meta-analysis was based on aggregated data from the included studies, which might not reflect the individual variability and complexity of the patients and the operations. Therefore, individual patient data meta-analysis might be more informative to compare the outcomes of CO and DC. Second, the evidence of pooled results was limited by the level of evidence of the included studies. Most of the included studies were retrospective, some of which lacked adjustments for confounders. Even though we used a meta-regression model and subgroup analysis to adjust the influence of baseline characteristic bias, considerable heterogeneity persisted. Thus, the generalizability and clinical value of pooled results might be compromised. To our knowledge, there is a scarcity of high-quality evidence, with only one published RCT (RESCUE-ASDH trial) on this topic to date^10^. Therefore, high-quality RCTs are appealed to provide more robust and conclusive evidence for the efficacy and safety of CO and DC. Third, the mortality data were extracted directly from the papers, which limited our ability to discern mortality at different time points. Directly conducting a pooled analysis would introduce bias into the results. Fourth, we failed to investigate overall complications, given the lack of relevant information. We focused solely on reoperation rates.

## Conclusion

In summary, our meta-analysis of existing evidence indicated DC had higher mortality and poorer neurological outcomes than CO in ASDH. The reoperation rates were similar between the two procedures. However, when baseline severity was adjusted to be equivalent, the choice between CO and DC did not impact patient prognoses. These findings emphasize the importance of baseline severity in interpreting outcomes and suggest that personalized treatment decisions, considering both the potential benefits and risks of CO and DC for specific patient profiles, may be crucial for optimal management of ASDH.

## Ethical approval

This is a systematic review and meta-analysis thus ethical approval is not required.

## Consent

No consent is required for this meta-analysis.

## Sources of funding

None.

## Author contribution

H.L., Y.Y., and W.G.: data curation; H.L., Y.S., and H.W.: formal analysis; C.Z. and Y.J.: investigation; W.G., Y.S., and H.W.: methodology; H.L., Y.Y., and C.Z.: software; H.L.: visualization; H.L., Y.Y., and W.G.: writing – original draft. All authors contributed in writing – review and editing and conceptualization.

## Conflicts of interest disclosure

The authors declares no conflicts of interest.

## Research registration unique identifying number (UIN)

This is a systematic review which has been registered in PROSPERO (CRD42023481857).

## Guarantor

Can Zhu: zhucanmedical@163.com. Yuwen Jiang: 1957449341@qq.com.

## Data availability statement

Ethical approval for this study is not required since it is a systematic review and meta-analysis.

## Provenance and peer review

Not commissioned, externally peer-reviewed.

## Presentation

None.

## Assistance with the study

None.

## Supplementary Material
