# Supplementary material for: Comparison of craniotomy and decompressive craniectomy for acute subdural hematoma: a meta-analysis of comparative study
Source: Int J Surg. 2024 May 13;110(8):5101–11. doi: 10.1097/JS9.0000000000001590 (PMC11326010; doi:10.1097/JS9.0000000000001590)
Supplement: Supplementary file 2 [file js9-110-5101-s002.docx]

**Identification of studies via databases**

Records removed before screening:

Duplicate records removed

(n = 257)

Records identified from:

PubMed (n = 247)

Embase (n = 330)

Web of Science (n = 229)

Cochrane Library (n = 25)

**Identification**

Records excluded by title and abstract

(n = 554)

Records screened

(n = 574)

**Screening**

Reports assessed for eligibility

(n = 20)

Reports excluded:

Reports including traumatic brain injuries (n = 3)

Reviews (n = 2)

Studies included in review

(n = 15)

**Included**
