# Supplementary material for: Comparison of craniotomy and decompressive craniectomy for acute subdural hematoma: a meta-analysis of comparative study
Source: Int J Surg. 2024 May 13;110(8):5101–11. doi: 10.1097/JS9.0000000000001590 (PMC11326010; doi:10.1097/JS9.0000000000001590)
Supplement: Supplementary file 4 [file js9-110-5101-s004.docx]

**Supplement Table 1. Search strategy**

| Database | Strategy |
| --- | --- |
| PubMed | (decompressive craniectomy) AND craniotomy AND (subdural hematoma OR subdural hemorrhage) AND ("1900/01/01"[Date - Publication] : "2023/10/01"[Date - Publication]) |
| Web of Science | (decompressive craniectomy) AND craniotomy AND (subdural hematoma OR subdural hemorrhage) AND DOP=(1900-01-01/2023-10-01) |
| Embase | #1: decompressive craniectomy  #2: craniotomy  #3: #1 AND #2  #3: subdural hematoma  #4: subdural hemorrhage  #5: #3 OR #4  #6: #3 AND #5 AND [01-01-1900]/sd NOT [02-10-2023]/sd |
| Cochrane Library | #1: decompressive craniectomy  #2: craniotomy  #3: subdural hematoma  #4: subdural hemorrhage  #5: #1 AND #2  #6: #3 OR #4  #7: #5 AND #6  with Cochrane Library publication date Between Jan 1900 and Oct 2023 |

Full Search strategy for PubMed (example)

("decompressive craniectomy"[MeSH Terms] OR ("decompressive"[All Fields] AND "craniectomy"[All Fields]) OR "decompressive craniectomy"[All Fields]) AND ("craniotomy"[MeSH Terms] OR "craniotomy"[All Fields] OR "craniotomies"[All Fields]) AND ("subdural haematoma"[All Fields] OR "hematoma, subdural"[MeSH Terms] OR ("hematoma"[All Fields] AND "subdural"[All Fields]) OR "subdural hematoma"[All Fields] OR ("subdural"[All Fields] AND "hematoma"[All Fields]) OR ("subdural haemorrhage"[All Fields] OR "hematoma, subdural"[MeSH Terms] OR ("hematoma"[All Fields] AND "subdural"[All Fields]) OR "subdural hematoma"[All Fields] OR ("subdural"[All Fields] AND "hemorrhage"[All Fields]) OR "subdural hemorrhage"[All Fields])) AND 1900/01/01:2023/10/01[Date - Publication]
