# Supplementary material for: Comparison of craniotomy and decompressive craniectomy for acute subdural hematoma: a meta-analysis of comparative study
Source: Int J Surg. 2024 May 13;110(8):5101–11. doi: 10.1097/JS9.0000000000001590 (PMC11326010; doi:10.1097/JS9.0000000000001590)
Supplement: Supplementary file 5 [file js9-110-5101-s005.pdf]

Supplement Figure 1. Bubble plot for the meta-regression model.

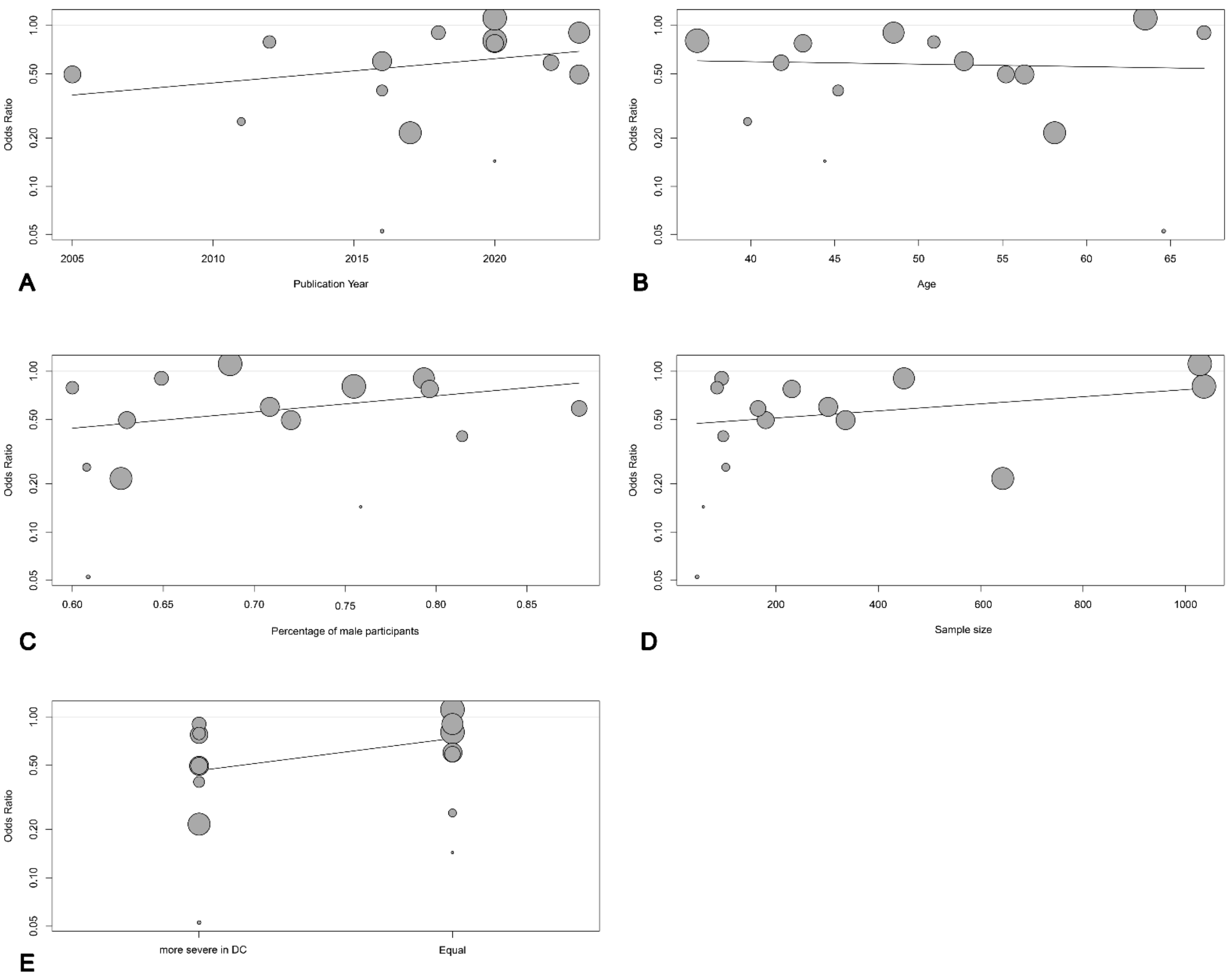

Bubble plots for the meta-regression results. (A) Effect size and publication year; (B) Effect size and mean age; (C) Effect size and percentage of male participants; (D) Effect size and sample size; (E) Effect size and comparability of severity, DC, decompressive craniectomy.
